# Would the Removal of Voluntary Iron Fortification Put Vulnerable Populations at Risk? Modelling the Risk of Inadequate and Excess Iron Intakes in Children in Ireland

**DOI:** 10.3390/nu18132144

**Published:** 2026-07-02

**Authors:** Laura Kehoe, Janette Walton

**Affiliations:** Department of Biological Sciences, Munster Technological University, T12 P928 Cork, Ireland; janette.walton@mtu.ie

**Keywords:** iron, fortification, voluntary fortification, iron intakes, children, safe maximum levels

## Abstract

**Background/Objectives**: Iron is an essential element for human health, with natural sources and fortified foods being the main contributors to intakes. In the context of setting safe maximum levels (SMLs) in food supplements and fortified foods in the EU, it is necessary to understand the current role of fortified foods in the diet and the potential impact of any regulatory changes. This study used three modelling scenarios to investigate the impact of removing voluntary iron fortification of current iron-fortified foods on iron intakes in children. **Methods**: Data were based on the Irish National Children’s Food Survey II. The modelling scenarios were as follows: 1. removal of iron from all fortified foods; 2. removal of iron from fortified foods excluding ready-to-eat cereals (RTECs); and 3. removal of iron from fortified RTECs only. Usual intakes of iron, the prevalence of inadequate intakes, and risk of excess intakes were examined at baseline and for each scenario for the total population and consumers only. **Results**: Removing the iron-fortified component from all iron-fortified foods/RTECs only significantly increased the prevalence of inadequate intakes of iron from 20% to 50%, with significantly higher proportions of females (55–61%) having inadequate intakes compared to males (37–44%). There was negligible risk of excess iron intake at baseline and no further impact from any of the three scenarios. **Conclusions**: This study showed that removing voluntary iron fortification could carry a significant nutritional risk and should be carefully evaluated to ensure that the iron status of vulnerable population groups is not negatively impacted.

## 1. Introduction

Iron is an essential element in human health, serving as a crucial cofactor in a wide array of biochemical processes. Beyond its well-established role in blood health through haemoglobin production and oxygen transport, iron is integral to cellular energy production, DNA synthesis, immune function, and the regulation of oxidative stress [1]. Iron deficiency anaemia (IDA) is recognised as the most common nutritional deficiency globally, affecting over 30% of the population [2]. While IDA is most prevalent in younger children and women, it is estimated that 9% of school-aged children globally have IDA, with prevalence estimates for Europe of 2–3% corresponding to over 2 million children impacted by this nutritional deficiency [2,3].

Across Europe, within national dietary surveys, it has been estimated that 20–40% of school-aged children have low iron intakes, and while these low intakes may not consistently correspond to clinical outcomes of IDA, low iron intakes or iron deficiency (with or without anaemia) can contribute to negative health outcomes impacting children’s attention, learning and physical capacity during this crucial period of growth and development [4,5,6,7]. The key sources of iron among all ages in Europe (including school-aged children) have been reported as natural sources (e.g., meat and meat dishes, cereal products) and fortified foods including those both voluntarily fortified (e.g., ready-to-eat cereals (RTECs)) and those with mandatory fortification (e.g., bread produced from fortified flour) with minimal contributions from food supplements [4,5,8,9]. The consumption of fortified foods in particular (both those voluntarily and mandatorily fortified) have been shown to contribute to improved iron intakes among all ages globally, with school-aged children often reported amongst the highest consumers of fortified foods in population groups [10,11,12,13]. However, balancing the benefits of improved nutrient intakes from fortified foods (or food supplements) with the potential risk of increasing intakes above upper limits is continually at the forefront of public health policy and food safety assessments.

Within the European Union (EU), the addition of vitamins and minerals to foods has been regulated through European Commission (EC) regulation No. 1925/2006 since 2007 [14]. This regulation has provided for the setting of safe maximum levels (SMLs) for the addition of vitamins and minerals to foods and food supplements, and while several models for the setting of SMLs have been proposed, no harmonised SMLs have yet been implemented at an EU level [15,16,17,18,19]. However, in recent years, the EC has set out to introduce SMLs for food supplements and fortified foods based on a re-evaluation of tolerable upper daily intakes (ULs) by the EFSA [20]. In the absence of sufficient data on which to set a UL for iron, based on established evidence that systemic iron overload leads to organ toxicity, the EFSA have set a safe level of intake (SI) based on the prevention of black stools from large amounts of unabsorbed iron in the gut [21]. However, preceding the setting of these SMLs by the EC, some countries have set out to provide their own recommendations at a national level intended as a scientific basis for the discussion of the setting of harmonised SMLs at the EU level, e.g., the German Federal Institute for Risk Assessment (BfR) updated their recommendations for maximum levels of vitamins and minerals in food supplements and fortified foods in 2021 [22]. These maximum recommended levels were outlined with the intention of limiting nutrient intake from fortified foods and food supplements to ensure a significant additional nutrient intake for those in need, whilst protecting the majority of the population with adequate intakes from an excessive intake. For iron, the BfR has outlined two potential options for the addition of iron to fortified foods, which are 1. no addition to any foods, or 2. limited addition to ‘breakfast cereals’, and set a maximum level conforming to current fortification practices [22].

Given the important contribution of fortified foods to current iron intakes among children (and other population groups), it is important to consider the potential impact on iron intakes and adequacy in this vulnerable age group if restrictions were to be placed on the voluntary addition of iron to foods or on the levels permitted. The National Children’s Food Survey II (NCFS II) in Ireland has collected detailed data on the food consumption of children aged 5–12 years at brand level, allowing for the estimation of nutrient intakes from all dietary sources, natural food sources, added nutrients in foods, and from food supplements [4]. Similar to findings from other national dietary surveys [23,24], RTECs were shown to be important contributors to iron intakes among this age group (up to 30%) which prompts the question of the potential impact on iron intakes for this population group if regulations surrounding fortified foods were to change [4].

Therefore, the aim of this study was to use the BfR example for iron and add to the evidence base for the discussion of setting SMLs at an EU level using the NCFS II data as an example. Specifically, this study aimed to investigate the impact of removing voluntary iron fortification of current iron-fortified foods on iron intakes, adequacy and excess in children based on three modelling scenarios: 1: removal of iron fortification from all iron fortified foods; 2: removal of iron fortification from all iron fortified foods excluding RTECs; and given the important contribution of RTECs to current iron intakes in this population group, 3: removal of iron fortification from RTECs only (excluding all other iron-fortified foods).

## 2. Materials and Methods

### 2.1. Study Sample

Analyses for the present study are based on data from the National Children’s Food Survey II (NCFS II), which was a cross-sectional food consumption survey conducted in the Republic of Ireland in the period 2017–18 by the Irish Universities Nutrition Alliance (IUNA) (www.iuna.net) to establish a database of habitual food and beverage consumption in a nationally representative sample of children aged 5–12 years (*n* 600) in Ireland. A detailed methodology for the NCFS II has previously been described [4,25] and an overview of the methods relevant to this study is outlined below.

Briefly, the data collection phase of the NCFS II was carried out between April 2017 and May 2018, providing a seasonal balance to the data collection. A quota sampling approach was adopted using data from the 2016 Census to achieve a nationally representative sample of 600 children (males: 300, females: 300) [26]. The study was conducted according to the guidelines laid down in the declaration of Helsinki and all procedures involving human participants were approved by the Clinical Research Ethics Committee of the Cork Teaching Hospitals, University College Cork and the Human Ethics Research Committee of University College Dublin (Ref: ECM 4 (aa) 07/02/17). Written informed consent was obtained from children and their parents/guardians. Demographic analysis of the sample has shown it to be representative of children in Ireland with respect to age-group, sex and geographical location when compared to Census 2016 data [26]. However, the final sample contained a higher proportion of children of professional workers and a lower proportion of children of semi-skilled and unskilled workers than the national population, and all data presented in this manuscript have been weighted to account for these differences.

### 2.2. Food and Beverage Consumption Data and Estimation of Nutrient Intakes

Food and beverage intake data (including food supplements) were collected at brand level using a 4-day weighed food record. For all participants, the study period included at least one weekend day. Participants were provided with a food diary and digital food scales (Tanita KD-400, Tanita Ltd., Tokyo, Japan) and asked to record detailed information regarding the amount, type and brand of all foods, beverages and food supplements consumed, as well as the amount of any leftovers. Details of recipes of composite dishes were also recorded. Participants were provided with packaging collection bags to retain the food label packaging of all foods, beverages and food supplements consumed during the recording period. Researchers made three visits to the participants’ homes over the survey period: an initial training visit to demonstrate how to complete the food diary and use the weighing scales; a second visit 24–36 h into the recording period to review the diary and clarify details regarding specific food descriptors and quantities; and a final visit one or two days after the recording period to review the last days of the diary and to collect the food diary and food scales.

The majority of foods and beverages were weighed by the participant or their parent/guardian directly on the digital food scales (76%) and a further 11% of weights were derived from manufacturers’ information on product labels. The remaining foods and beverages were quantified using photographic food atlases (7%) [27], standard portion sizes (3%) [28,29], household measures (1%) and estimates based on the child’s previous eating patterns (used only when no other quantification method was appropriate) (2%). For all methods of quantification, leftovers were accounted for, and the weight of the food consumed was calculated.

Nutritics^©^ software (Research Edition) (Dublin, Ireland) was used to estimate nutrient intakes from food, beverage and food supplement intakes using data from McCance and Widdowson’s *The Composition of Foods*, seventh edition and sixth edition (for a small number of foods) [30,31]. During the survey, modifications were made to the food composition database to include recipes of composite dishes, food supplements, fortified foods and generic Irish foods that were commonly consumed. All food label packaging collected throughout the survey was photographed to capture information from the ingredient list and nutritional labels. Where packaging was not available in the participant’s home, the researchers located the item in the relevant retail outlet and photographed it.

### 2.3. Identification of Iron-Containing Food Supplements

Iron-containing food supplements were identified as those that had iron present in the ingredient list. Consumers of iron-containing food supplements were defined as those who consumed an iron-containing food supplement on any day during the survey period (4 days).

### 2.4. Identification of Iron-Fortified Foods and Their Consumers

Iron-fortified foods were identified as those that had iron present in the ingredient list (regardless of content per 100 g). RTECs were defined as all RTECs including muesli/granola, etc., but excluding porridge and hot oat cereals. Consumers of iron-fortified foods and consumers of iron-fortified RTECs were defined as those who consumed any amount of an iron-fortified food or an iron-fortified RTEC on any day during the survey period (regardless of frequency of consumption or portion size consumed). Of the foods fortified with iron in the NCFS II, approximately 66% were RTECs, followed by breakfast-/cereal-type bars (15%), hot oat cereals (6%), milks and milk-based beverages (5%), with the remaining 7% consisting of retail savoury products, biscuits/crackers and composite dishes made with iron fortified ingredients.

### 2.5. Identification of the Naturally Occurring Iron in Iron-Fortified Foods

Natural levels of iron present in iron-fortified foods were identified in accordance with previous studies of national dietary surveys in Ireland by obtaining food composition data for an unfortified equivalent of the food, or, if an unfortified equivalent was not available, based on data previously provided by manufacturers during the IUNA national dietary surveys [30,32,33,34,35].

### 2.6. Baseline Intakes and Modelling Scenarios

Baseline data reflects the intakes of iron based on actual dietary patterns and food composition as per the NCFS II. The modelling scenarios included:

1. Model 1 (FeFortFoods_all): Removal of iron fortification from all iron-fortified foods
2. Model 2 (FeFortFoods_exclRTEC): Removal of iron fortification from all iron-fortified foods excluding RTECs
3. Model 3 (FeFortRTEC_exclother): Removal of iron fortification from RTECs only (excluding all other iron-fortified foods)

All other nutrient composition remained unchanged in the baseline and all modelling scenarios.

### 2.7. Estimation of Usual Iron Intakes

Usual intake distributions of iron from all sources (food, beverages and food supplements) and from food sources only (food and beverages, excluding food supplements) at baseline and for each of the three modelling scenarios were estimated for all children aged 5–12 years, for consumers of iron-fortified foods, and for consumers of iron-fortified RTECs using the validated National Cancer Institute (NCI) method [36] which accounts for both inter- and intra-person variance. The NCI-method has been implemented in SAS macros (version 2.1) which were downloaded from www.riskfactor.cancer.gov/diet/usualintakes/macro.html (date of download/accessed: 1 July 2015). For these analyses, the covariates used were sex (male/female) and age group (5–8 y/9–12 y) in line with previous data analyses from the NCFS II [4].

### 2.8. Adequacy of Iron Intakes

The prevalence of inadequate intakes of iron was estimated using the estimated average requirement (EAR) from the EFSA as a cut point [37]. The EAR is the level of (nutrient) intake estimated to meet the requirements of 50% of a population group [38]. This method has been shown to be effective in obtaining a realistic estimate of the prevalence of dietary inadequacy [39]. As under-reporting of food consumption can result in an overestimation of the prevalence of inadequacy in a population group [40], under-reporters (URs) were identified and excluded from these analyses (19.5% of total sample). URs were identified using Goldberg’s cut-off2 criterion updated by Black (which evaluates the ratio of energy intake to basal metabolic rate (EI:BMR) against age-specific energy cut-offs based on physical activity levels) [41,42,43,44].

### 2.9. Risk of Excessive Intake of Iron

Whilst the risk of excessive intake of micronutrients is typically evaluated using the tolerable upper intake level (UL) (maximum level of total chronic daily intake of a nutrient (from all sources) judged to be unlikely to pose a risk of adverse health effects to humans [45]), a recent review of the evidence to establish a UL for iron intakes by the EFSA concluded that there was insufficient evidence to establish a UL and instead set a Safe Level of intake (SI) [21]; the proportion of children with intakes above this SI were calculated within this study.

### 2.10. Statistical Analysis

Statistical analysis was carried out using SPSS^©^ for Windows™ Version 28.0. Differences in intakes of iron between sexes (males, females) were assessed using independent sample *t*-tests. Differences in intakes of iron between baseline and the modelled scenarios were assessed using paired sample *t*-tests regardless of normality (due to the large sample size). As sample size increases so does the robustness of *t*-tests to identify deviations from normality; thus, parametric tests are recommended for large samples [46]. Differences in the prevalence of inadequate intakes of iron (proportion of children with intakes below the EAR) between baseline and the modelled scenarios and between sexes (males, females) were assessed using Chi-square tests. To minimise type 1 errors (as a result of multiple testing), the Bonferroni adjustment was used by dividing the alpha level (0.05) by the number of comparisons, with intakes considered to be significantly different from each other if *p* < 0.001 [46]. However, due to the large sample in this study, even a small difference between group means was highly statistically significant; thus, greater emphasis was placed on a descriptive—rather than a formal—statistical analysis of the data.

## 3. Results

Table 1 presents the distribution of iron intakes (mg), the proportion of the population with intakes below the EAR (excluding energy-under reporters) (%) and the proportion with intakes above the SI (%) from all sources (including food supplements) and food sources only (excluding food supplements) in children aged 5–12 years in Ireland in the total population of children, among consumers of iron-fortified foods and among consumers of iron-fortified RTECs based on actual intakes (baseline) and after modelling the removal of the iron-fortified component of iron-fortified foods as per the three modelling scenarios.

The proportion of children consuming any iron-fortified food was 82% and the proportion consuming iron-fortified RTECs was 78%. Among the total population of children, the mean intake of iron from all sources (including nutritional supplements) at baseline was 9.0 ± 2.4 mg, with significantly lower intakes observed following all modelling scenarios which restricted iron fortification: FeFortFoods_all (7.0 ± 1.8 mg), FeFortFoods_exclRTEC (8.9 ± 2.4 mg) and FeFortRTEC_exclother (7.1 ± 1.8 mg). Similarly, among consumers of iron-fortified foods, the mean intake of iron from all sources was significantly lower in all modelling scenarios—FeFortFoods_all (6.9 ± 1.7 mg), FeFortFoods_exclRTEC (9.3 ± 2.4 mg) and FeFortRTEC_exclother (7.1 ± 1.7 mg)—than baseline (9.5 ± 2.3 mg), and for consumers of iron-fortified RTECs, the mean intake of iron from all sources at baseline was (9.5 ± 2.3 mg) with significantly lower intakes observed following all modelling scenarios: FeFortFoods_all (6.9 ± 1.7 mg), FeFortFoods_exclRTEC (9.4 ± 2.4 mg) and FeFortRTEC_exclother (7.0 ± 1.7 mg). As the proportion of children using an iron containing supplement was low (6%;), similar findings were found for intakes from food sources only (as with all sources) with mean intakes of iron significantly lower in each of the modelling scenarios compared to baseline for the total population of children, consumers of iron-fortified foods and consumers of iron-fortified RTECs.

**Figure 1:**
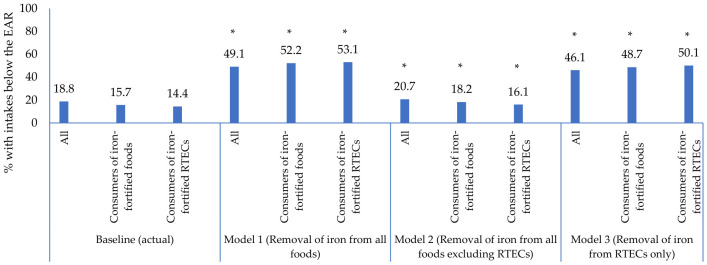
Prevalence of inadequate iron intakes (% < EAR) at baseline and across each modelling scenario for the total population, consumers of iron-fortified foods and consumers of iron-fortified RTECs. * Statistically different (*p* < 0.001) from % below the EAR at baseline via chi-square tests and adjusted for multiple testing.

The proportion of children with iron intakes below the EAR from all sources at baseline was 19%, and this was significantly higher in FeFortFoods_all (49%), FeFortFoods_exclRTEC (21%) and FeFortRTEC_exclother (46%) (Figure 1). Similarly, the proportion of children with iron intakes below the EAR from all sources was significantly higher in each modelling scenario compared to baseline for consumers of iron-fortified foods (baseline: 16%, FeFortFoods_all: 52%, FeFortFoods_exclRTEC: 18%, FeFortRTEC_exclother: 49%) and consumers of iron fortified RTEC (baseline: 14%, FeFortFoods_all: 53%, FeFortFoods_exclRTEC: 16%, FeFortRTEC_exclother: 50%).

The proportion of children with intakes above the SI from all sources at baseline was negligible (0.2%) but was significantly lower in all three modelling scenarios (<0.1%, for each), with similar findings among consumers of iron-fortified foods and consumers of iron-fortified RTECs.

Table 2 presents the distribution of iron intakes (mg), the proportion of the population with intakes below the EAR (excluding energy-under reporters) (%) and the proportion with intakes above the SI (%) from all sources (including food supplements) and food sources only in children aged 5–12 years in Ireland by sex in the total population of children, among consumers of iron fortified foods and among consumers of iron fortified RTECs based on actual intakes (baseline) and after modelling the removal of the iron-fortified component of iron-fortified foods as per the three modelling scenarios.

At baseline, females had significantly lower mean intakes of iron from all sources compared to males, in the total population (males: 9.8 ± 2.5 mg; females: 8.4 ± 2.1 mg), among consumers of iron-fortified foods (males: 10.2 ± 2.4 mg; females: 8.8 ± 2.0 mg) and among consumers of iron-fortified RTECs (males: 10.2 ± 2.4 mg; females: 8.9 ± 2.0 mg). Similarly, females had significantly lower intakes of iron from all sources in each modelling scenario and from food sources only at baseline and in each modelling scenario.

Significantly higher proportions of females had iron intakes below the EAR from all sources and food sources only at baseline and in each modelling scenario. At baseline, up to 25% of females (total population, consumers of iron-fortified foods and consumers of iron-fortified RTECs) were at risk of inadequate iron intakes compared to up to 12% of males. In FeFortFoods_all, up to 61% of females were at risk of inadequate iron intakes compared to up to 44% of males. In FeFortFoods_exclRTEC, up to 28% of females were at risk of inadequate iron intakes compared to up to 13% of males. In FeFortRTEC_exclother, up to 58% of females were at risk of inadequate iron intakes compared to up to 41% of males.

## 4. Discussion

Given the importance of iron at all stages of the lifecycle and the potential regulatory changes surrounding food fortification, this study aimed to examine the potential impact of removing the iron-fortified component of iron-fortified foods using three modelling scenarios to provide an evidence base for the discussion of the setting of SMLs for the addition of vitamins and minerals to foods at an EU level. More specifically, this study examined the impact of removing the iron-fortified component of all iron-fortified foods, all iron-fortified foods excluding RTECs or iron-fortified RTECs only on the intake of iron in children aged 5–12 years in Ireland including the prevalence of inadequate intakes and risk of excess intakes. The main finding was that removing the iron-fortified component from all iron-fortified foods or from RTECs only would significantly increase the prevalence of inadequate intakes of iron to approximately one half of all children compared to current levels (approximately one fifth), with significantly higher proportions of females (55–61%) having inadequate intakes compared to males (37–44%) in all modelling scenarios. However, removing the iron-fortified component from all foods excluding RTECs would have little impact on the prevalence of inadequate intakes in children compared to current levels, albeit with significant proportions (approximately one-fifth) remaining with inadequate iron intakes. All scenarios showed negligible risk of iron intakes above the SI.

In order to set SMLs for the fortification of foods with vitamins and minerals, it is essential to understand the current role of fortified foods in the diet and the potential impact of restrictions or changes to the legislation surrounding these foods on intakes and the risk of inadequate nutrient intakes in vulnerable population groups [19]. Furthermore, previously proposed models for the setting of SMLs have outlined the need to account for all potential sources of nutrient intakes within the diet (natural foods, fortified foods and food supplements) [15,16,17,18,19]. The NCFS II data allowed for this modelling exercise to examine the impact of full restrictions on fortified foods, various models of inclusion of iron-fortified foods and for each model to examine the intakes from all sources (all foods and food supplements) and from food sources only (excluding food supplements). Of the scenarios examined in this study, removal of the iron-fortified component from all iron-fortified foods or just from RTECs would substantially increase the prevalence of inadequate iron intakes in children from approximately one fifth to up to one half with a significantly higher prevalence amongst females (up to 61%) compared to males (up to 44%). These findings should be carefully considered, as presently in Ireland (a country with a long-standing, liberal policy on food fortification), significant proportions of children already have inadequate intakes of iron, which may have implications for cognitive and behavioural development at this age [4,47]. Further exacerbation of these high levels of inadequate intakes, particularly for females, would have additional implications for older girls due to the onset of menstruation, which may elevate the risk of low iron stores and IDA [4,47]. While there are no biochemical data of iron status available from the NCFS II, low iron status has been found for counterparts of this age group in the UK, with increasing prevalence in teenagers (particularly for females) [8]. A similar modelling study to ours in the US which examined three scenarios, baseline, zero fortification and optimised fortification, for a number of nutrients reported that the optimisation of RTEC fortification could be useful to minimise the proportion of the population with intakes below the EAR for all nutrients including iron across all age groups (1 y+) (0% at baseline or optimised fortification compared to 7% for zero fortification) [48].

In the current study, similar findings were observed across all scenarios whether examining intakes from all dietary sources (including food supplements) or from food sources only (excluding food supplements) which was to be expected given the low prevalence of iron supplement users (6%); thus, it can be observed that iron intakes in this population group are primarily driven by food intake. Similar to our study, a study in the UK examining the contribution of base diet, voluntary fortified foods and supplements to micronutrient intakes found that voluntary fortified foods (but not supplements) made a meaningful contribution to intakes of vitamin and minerals, without the risk of unacceptably high intakes, with fortified foods contributing up to 13% of total iron intake across all ages [10]. The same study also reported that voluntary fortified foods helped to reduce the prevalence of inadequate intakes for many nutrients including iron reducing the prevalence from 45% from base diet only to approximately 33% from base diet and fortified foods (~30% from base diet, fortified foods and supplements) [10]. Given the increasing emphasis on planetary health, in particular surrounding the advice to reduce the intakes of animal-based foods which are important natural sources of iron [49,50] and possible future restrictions in iron levels of voluntary fortified foods, the findings of this study indicate that any policy recommendations surrounding changes to the composition of the existing food supply should be carefully considered particularly in light of shifting dietary patterns amongst population groups.

At the forefront of setting SMLs for vitamins and minerals, it is of utmost importance to balance the benefits of improved nutrient intakes from fortified foods (and/or food supplements) with the potential risk of increasing intakes above upper limits/safe intakes. In the current study, each of the modelling scenarios for restricting the iron fortification of foods showed negligible risk of iron intakes above the SI, which is unsurprising given the negligible risk at baseline based on current dietary patterns among this population group, which has also been reflected in other national dietary survey data of both children and other population groups where the risk of excessive iron intakes are negligible (<1–3%) based on current dietary patterns across Europe and the developed world [51,52,53,54].

As this study focused on the modelling of iron intakes alone rather than the biochemical status of iron, it should be acknowledged that haem iron (from animal sources) is more bioavailable than non-haem iron (from plant sources); thus, the potential impact on biochemical iron status cannot be fully elucidated from this study. Nonetheless, this study has provided evidence on the impact of removing iron fortification from foods on intakes of iron in school-aged children, and the findings may have potential implications for other population groups, both in Ireland and globally. National dietary surveys of other population groups in Ireland and across the developed world have consistently shown that (iron-fortified) RTECs are key contributors to iron intakes in all age groups [23,24,55,56,57,58,59,60,61,62,63,64], and a recent systematic review including data from five different countries showed that RTECs provided up to 28% of daily iron intakes in the total population across all ages, and from 32 to 51% of daily iron intake in RTEC consumers only [56]. Any change to regulations around the fortification of foods could have implications not only for iron but for other nutrients that are routinely added to RTECs, or other nutrients which are largely obtained from fortified foods (e.g., vitamin D, folic acid) [56].

### Strengths and Limitations

The key strengths of this study include the nationally representative sample of children aged 5–12 years included in the NCFS II and the comprehensive dietary intake and food composition data (including brand level detail) which allowed for the estimation of naturally occurring and added iron in foods. Another important strength is the use of statistical modelling to estimate usual intakes of iron, resulting in a better estimate of the true distribution of usual intakes, therefore improving the estimates of the proportions of the population with intakes above or below a particular reference value (e.g., EAR or SI) which would otherwise be overestimated. Misreporting or under-reporting of food (energy) intake, is a known limitation with all dietary assessment; this issue was minimised by a high level of researcher–participant interaction (3–4 visits over the recording period). Additionally, the removal of URs from estimates of the prevalence of inadequacy provides a better representation of the scale of nutrient inadequacy. This study also examined all consumers of iron-fortified foods and RTECs regardless of frequency of consumption or amount consumed, which may limit the interpretation of findings to population-level implications rather than smaller subgroups (e.g., low-frequency consumers) and on the basis of modelling dietary intakes only may limit the extrapolation of findings to overall dietary intakes rather than iron status.

## 5. Conclusions

In summary, this study has shown that removing the iron-fortified component from all iron-fortified foods or from RTECs only would significantly increase the prevalence of inadequate intakes of iron to approximately one half compared to current levels (approximately one fifth), with significantly higher proportions of females (55–61%) having inadequate intakes compared to males (37–44%) in all modelling scenarios. This study used iron as an example to investigate the potential impact and unintended consequences of removing fortification from specific foods and contributes to the evidence base to support discussions surrounding the setting of SMLs for vitamins and minerals at an EU level. Beyond the setting of SMLs at an EU level, this study has shown the role that current levels of voluntary fortification play in protecting against inadequate iron intakes among children. In light of evolving dietary patterns driven by planetary health concerns and ongoing reformulation policies, continual monitoring of food consumption patterns and dietary intakes (from both natural and fortified food sources and supplements) is essential to ensure that the nutritional status of the population is not negatively impacted and to continue to inform appropriate public health strategies across the lifecycle.

## Figures and Tables

**Table 1:** Distribution of iron intakes (mg), the proportion of the population with intakes below the Estimated Average Requirement (EAR) [37] (excluding energy-under reporters ^‡^) (%) and the proportion with intakes above the Safe Level of Intake (SI) [21] (%) from all sources ^†^ (including food supplements) and food sources ^†^ only (excluding food supplements) in children aged 5–12 years in Ireland in the total population of children, among consumers of iron-fortified foods and among consumers of iron-fortified ready-to-eat-cereals (RTECs) based on current intakes (baseline) and after modelling the removal of the iron-fortified component of iron-fortified foods per the three modelling scenarios.

	Baseline (Actual)			Model 1 (Removal of Iron from All Foods)			Model 2 (Removal of Iron from All Foods Excluding RTECs)			Model 3 (Removal of Iron from RTECs Only)		
	All	Consumers of Iron-Fortified Foods	Consumers of Iron-Fortified RTECs	All	Consumers of Iron-Fortified Foods	Consumers of Iron-Fortified RTECs	All	Consumers of Iron-Fortified Foods	Consumers of Iron-Fortified RTECs	All	Consumers of Iron-Fortified Foods	Consumers of Iron-Fortified RTECs
	*n* 600	*n* 493	*n* 466	*n* 600	*n* 493	*n* 466	*n* 600	*n* 493	*n* 466	*n* 600	*n* 493	*n* 466
	mg/d											
All sources ^†^												
Mean	9.0	9.5	9.5	7.0 *	6.9 *	6.9 *	8.9 *	9.3 *	9.4 *	7.1 *	7.1 *	7.0 *
SD	2.4	2.3	2.3	1.8	1.7	1.7	2.4	2.4	2.3	1.8	1.7	1.7
P5	5.5	6.1	6.2	4.4	4.4	4.5	5.4	5.9	6.0	4.6	4.6	4.6
P25	7.3	7.8	7.9	5.7	5.7	5.7	7.2	7.6	7.7	5.8	5.9	5.8
P50	8.8	9.2	9.3	6.8	6.7	6.7	8.6	9.0	9.1	6.9	6.9	6.8
P75	10.5	10.9	10.9	8.0	7.9	7.9	10.3	10.7	10.7	8.2	8.1	8.0
P95	13.5	13.7	13.6	10.2	10.0	9.9	13.3	13.5	13.5	10.4	10.2	10.0
	%											
% < EAR [37] ^‡^	18.8	15.7	14.4	49.1 *	52.2 *	53.1 *	20.7 *	18.2 *	16.1 *	46.1 *	48.7 *	50.1 *
% > SI [21]	0.2	0.2	0.2	0.0*	0.0*	0.0 *	0.1 *	0.2 *	0.1 *	0.0 *	0.0 *	0.0 *
	mg/d											
Food sources ^†^												
Mean	8.8	9.2	9.2	6.7 *	6.6 *	6.6 *	8.6 *	9.0 *	9.1 *	6.8 *	6.8 *	6.8 *
SD	2.2	2.1	2.1	1.5	1.4	1.4	2.2	2.2	2.1	1.5	1.4	1.4
P5	5.5	6.1	6.2	4.6	4.6	4.6	5.4	5.9	6.1	4.7	4.7	4.7
P25	7.2	7.7	7.8	5.7	5.6	5.6	7.0	7.5	7.6	5.8	5.8	5.8
P50	8.5	9.0	9.0	6.6	6.5	6.5	8.4	8.8	8.9	6.7	6.7	6.6
P75	10.1	10.5	10.5	7.6	7.5	7.4	9.9	10.3	10.4	7.8	7.7	7.6
P95	12.8	13.0	13.0	9.3	9.2	9.1	12.6	12.9	12.9	9.5	9.4	9.2
	%											
% < EAR [37] ^‡^	20.5	17.2	15.6	54.0 *	57.2 *	57.6 *	22.9 *	19.8 *	17.6 *	50.8 *	53.2 *	54.6
% > SI [21]	0.1	0.1	0.1	0.0 *	0.0 *	0.0	0.0 *	0.1 *	0.0 *	0.0 *	0.0 *	0.0 *

Abbreviations: RTEC, ready-to-eat cereal; M, males; F, females; mg, milligram; d, day; %, percentage; SD, standard deviation; P, percentile; <, below; EAR, estimated average requirement; >, above; SI, safe level of intake. Note: All includes non-consumers and consumers of any iron-fortified food; Consumers of iron-fortified foods were defined as any participant who consumed an iron-fortified food at least once over the 4-day recording period; Consumers of iron-fortified RTEC were defined as any participant who consumed an iron-fortified RTEC at least once over the 4-day recording period. ^†^ All sources refers to all sources including foods and food supplements; Food sources refers to food sources only (excludes food supplements), * Statistically different (*p* < 0.001) from intake at baseline via paired samples *t*-tests and % below the EAR/% above the SI at baseline via chi-square tests and adjusted for multiple testing. EAR: EFSA, 2015 [37]; 5–6 y (5 mg/d), 7–11 y (8 mg/d), 12 y males (8 mg/d), 12 y females (7 mg/d), ^‡^ Excludes energy-under reporters (19.5%), SI: EFSA, 2024 [21]; 5–6 y (15 mg/d), 7–10 y (20 mg/d), 11–12 y (30 mg/d).

**Table 2:** Distribution of iron intakes (mg), the proportion of the population with intakes below the Estimated Average Requirement (EAR) [37] (excluding energy-under reporters ^‡^) (%) and the proportion with intakes above the Safe Level of Intake (SI) [21] (%) from all sources ^†^ (including food supplements) and food sources ^†^ only (excluding food supplements) in children aged 5–12 years in Ireland by sex in the total population of children, among consumers of iron-fortified foods and among consumers of iron-fortified ready-to-eat-cereals (RTECs) based on current intakes (baseline) and after modelling the removal of the iron-fortified component of iron-fortified foods per the three modelling scenarios.

	Baseline (Actual)						Model 1 (Removal of Iron from All Foods)						Model 2 (Removal of Iron from All Foods Excluding RTEC)						Model 3 (Removal of Iron from RTEC Only)					
	All		Consumers of Iron-Fortified Foods		Consumers of Iron-Fortified RTECs		All		Consumers of Iron-Fortified Foods		Consumers of Iron-Fortified RTECs		All		Consumers of Iron-Fortified Foods		Consumers of Iron-Fortified RTECs		All		Consumers of Iron-Fortified Foods		Consumers of Iron-Fortified RTECs	
	M	F	M	F	M	F	M	F	M	F	M	F	M	F	M	F	M	F	M	F	M	F	M	F
	*n* 300	*n* 300	*n* 244	*n* 249	*n* 230	*n* 236	*n* 300	*n* 300	*n* 244	*n* 249	*n* 230	*n* 236	*n* 300	*n* 300	*n* 244	*n* 249	*n* 230	*n* 236	*n* 300	*n* 300	*n* 244	*n* 249	*n* 230	*n* 236
	mg/d																							
All sources ^†^																								
Mean	9.8	8.4 *	10.2	8.8 *	10.2	8.9 *	7.5	6.5 *	7.4	6.4 *	7.3	6.4 *	9.6	8.2 *	10.0	8.6 *	10.1	8.7 *	7.7	6.6 *	7.6	6.6 *	7.5	6.6 *
SD	2.5	2.1	2.4	2.0	2.4	2.0	1.9	1.5	1.8	1.5	1.8	1.4	2.5	2.1	2.5	2.0	2.4	2.0	1.9	1.5	1.8	1.5	1.8	1.5
P5	6.1	5.2	6.6	5.8	6.6	5.9	4.8	4.2	4.8	4.2	4.8	4.3	5.9	5.1	6.3	5.6	6.5	5.8	5.0	4.3	4.9	4.4	4.9	4.4
P25	8.0	6.8	8.5	7.4	8.5	7.5	6.2	5.4	6.1	5.4	6.1	5.4	7.8	6.7	8.2	7.2	8.3	7.3	6.4	5.5	6.3	5.5	6.2	5.6
P50	9.6	8.2	10.0	8.6	10.0	8.7	7.3	6.3	7.2	6.3	7.1	6.3	9.4	8.0	9.8	8.4	9.8	8.6	7.5	6.5	7.5	6.5	7.3	6.5
P75	11.3	9.7	11.7	10.0	11.7	10.1	8.7	7.4	8.6	7.3	8.4	7.3	11.2	9.5	11.6	9.8	11.6	10.0	8.8	7.5	8.7	7.5	8.6	7.5
P95	14.3	12.2	14.6	12.3	14.5	12.4	10.8	9.2	10.7	9.0	10.5	9.0	14.1	12.0	14.5	12.1	14.4	12.2	11.0	9.4	10.9	9.2	10.6	9.2
	%																							
% < EAR [37] ^‡^	11.9	25.1 *	9.2	21.5 *	8.9	19.3 *	39.4	58.0 *	42.2	61.2 *	44.1	61.2 *	13.2	27.5 *	11.2	24.6 *	10.1	21.7 *	36.5	54.9 *	38.9	57.6 *	41.4	57.9 *
% > SI [21]	0.3	0.1 *	0.3	0.1 *	0.3	0.1 *	0.0	0.0	0.0	0.0	0.0	0.0	0.2	0.1 *	0.3	0.0 *	0.2	0.0 *	0.0	0.0	0.0	0.0	0.0	0.0
	mg/d																							
Food sources ^†^																								
Mean	9.4	8.2 *	9.8	8.6 *	9.9	8.7 *	7.2	6.3 *	7.1	6.2 *	7.0	6.2 *	9.3	8.0 *	9.6	8.4 *	9.7	8.5 *	7.3	6.4 *	7.3	6.4 *	7.1	6.4 *
SD	2.3	1.9	2.3	1.8	2.2	1.8	1.5	1.2	1.5	1.2	1.4	1.2	2.3	1.9	2.3	1.8	2.2	1.8	1.5	1.3	1.5	1.2	1.4	1.2
P5	6.0	5.3	6.5	5.8	6.6	5.9	4.9	4.4	4.8	4.4	4.8	4.5	5.9	5.2	6.2	5.6	6.4	5.8	5.0	4.5	5.0	4.5	5.0	4.6
P25	7.8	6.8	8.2	7.3	8.3	7.4	6.1	5.4	6.0	5.4	5.9	5.4	7.6	6.6	8.0	7.1	8.1	7.3	6.2	5.5	6.2	5.5	6.1	5.6
P50	9.2	8.0	9.6	8.5	9.7	8.5	7.0	6.2	6.9	6.1	6.8	6.1	9.1	7.8	9.4	8.3	9.5	8.4	7.2	6.3	7.1	6.3	7.0	6.3
P75	10.8	9.3	11.3	9.8	11.2	9.8	8.1	7.0	8.0	7.0	7.9	7.0	10.7	9.2	11.1	9.6	11.1	9.7	8.3	7.2	8.2	7.2	8.1	7.1
P95	13.5	11.6	13.9	11.8	13.8	11.9	9.9	8.4	9.8	8.3	9.6	8.3	13.4	11.4	13.8	11.6	13.6	11.7	10.1	8.6	10.0	8.6	9.7	8.5
	%																							
% < EAR [37] ^‡^	13.1	27.3 *	10.6	23.2 *	9.7	20.9 *	44.2	63.0 *	47.3	66.2 *	48.7	65.7 *	14.8	30.2 *	12.6	26.5 *	11.1	23.4 *	41.2	59.6 *	43.5	62.0 *	46.0	62.4 *
% > SI [21]	0.1	0.0 *	0.1	0.0 *	0.1	0.0 *	0.0	0.0	0.0	0.0	0.0	0.0	0.1	0.0 *	0.1	0.0 *	0.1	0.0 *	0.0	0.0	0.0	0.0	0.0	0.0

Abbreviations: RTEC, ready-to-eat cereal; M, males; F, females; mg, milligram; d, day; %, percentage; SD, standard deviation; P, percentile; <, below; EAR, estimated average requirement; >, above; SI, safe level of intake. Note: All includes non-consumers and consumers of any iron-fortified food; Consumers of iron-fortified foods were defined as any participant who consumed an iron-fortified food at least once over the 4-day recording period; Consumers of iron-fortified RTEC were defined as any participant who consumed an iron-fortified RTEC at least once over the 4-day recording period. ^†^ All sources refers to all sources including foods and food supplements; Food sources refers to food sources only (excludes food supplements), * Statistically different (*p* < 0.001) from that of males within each scenario via independent samples *t*-tests and % below the EAR/% above the SI at baseline via chi-square tests and adjusted for multiple testing, EAR: EFSA, 2015 [37]; 5–6 y (5 mg/d), 7–11 y (8 mg/d), 12 y males (8 mg/d), 12 y females (7 mg/d), ^‡^ Excludes energy-under reporters (19.5%), SI: EFSA, 2024 [21]; 5–6 y (15 mg/d), 7–10 y (20 mg/d), 11–12 y (30 mg/d).

## Data Availability

Restrictions apply to the availability of these data. Data were obtained from the Irish Universities Nutrition Alliance and are available [from the authors/at www.iuna.net] with permission from the IUNA data access committee.

## Abbreviations

The following abbreviations are used in this manuscript:

SMLs	Safe Maximum Levels
RTECs	Ready-To-Eat Cereals
IDA	Iron Deficiency Anaemia
EU	European Union
EC	European Commission
UL	Tolerable Upper Intake Level
EFSA	European Food Safety Authority
BfR	German Federal Institute for Risk Assessment
NCFS II	National Children’s Food Survey II
EAR	Estimated Average Requirement
SI	Safe Level Of Intake
